# Role of percent peripheral tissue ablated on refractive outcomes following hyperopic LASIK

**DOI:** 10.1371/journal.pone.0170559

**Published:** 2017-02-02

**Authors:** George Fatseas, Fiona Stapleton, Patrick Versace

**Affiliations:** 1 School of Optometry and Vision Science, Faculty of Science, UNSW Australia, Kensington, New South Wales, Australia; 2 Vision Eye Institute, Bondi Junction, New South Wales, Australia; Bascom Palmer Eye Institute, UNITED STATES

## Abstract

**Objectives:**

To determine the effect of hyperopic laser in situ keratomileusis (H-LASIK) on corneal integrity, by investigating relationships between proportionate corneal tissue ablated and refractive outcomes at 3 months.

**Methods:**

18 eyes of 18 subjects treated with H-LASIK by Technolas 217c Excimer Laser were included in the study. Orbscan II Topography System was used to determine corneal volume and pachymetry 3mm temporally (3T). The volume of corneal tissue ablated was determined from the laser nomogram. Univariate associations between age, treatment, corneal volume, overall proportion of tissue removed, proportion of tissue removed at 3T, residual bed thickness at 3T and refractive outcomes 3 months post-LASIK were examined and independent factors associated with refractive outcomes determined using linear regression models.

**Results:**

At 3 months post-LASIK, the mean difference to expected refractive outcome was -0.20 ± 0.64 (Range -2.00 to +1.00). In univariate analysis, difference to expected refractive outcome was associated with proportion of tissue removed at 3T (P<0.01, r = -0.605) and total number of pulses (P< 0.05, r = -0.574). In multivariable analysis, difference to expected refractive outcome was associated with the proportion of tissue removed at 3T only.

**Conclusion:**

Subjects undergoing H-LASIK, may present as either over or under-corrected at 3 months. The proportion of tissue removed at 3T was the single significant determinant of this outcome, suggesting unexpected biomechanical alterations resulting in corneal steepening. Future hyperopic LASIK procedures could consider proportionate volume of corneal tissue removed at 3T in addition to laser nomograms to achieve improved refractive outcomes.

## Introduction

The cornea is comprised of 5 layers including the epithelium, Bowman’s layer, stroma, Descemet’s membrane and endothelium. It has various functions including protection, light transmission and refraction [1].

Being the scaffold for the refractive surface of the eye, any response to mechanical or biological injury will significantly influence the eye’s optical performance. One such intervention is laser in situ keratomileusis (LASIK), which is currently the most commonly performed refractive surgery for hyperopia [2, 3].

LASIK is effective in correcting myopia and hyperopia [4, 5], however, loss of effect can occur, being more common after hyperopic procedures [6, 7]. Age, degree of correction and pre-operative corneal curvature, are factors associated with the refractive outcomes following myopic LASIK [8–12]. The limited information available in hyperopic LASIK (H-LASIK) proposed pre-operative curvature and amount of correction to be associated with the refractive outcomes [13–15]. The degree of hyperopia corrected had a significantly negative outcome with corrections as low as +3.00 to +3.9 D showing higher rates of retreatments when compared to LASIK for low myopia [13]. Interestingly predictability and safety worsen with higher corrections [13].

In an effort to improve outcomes of hyperopic refractive surgery, Refractive lenticule extraction (ReLEx) using the Visumax femtosecond system was developed in 2008 [16], and further modified to small-incision lenticule extraction (SMILE), that required no flap. The lenticule that is created is extracted through a small arcuate incision [17]. Having no flap, this procedure could have benefits in less impact on corneal biomechanical stability [18]. Blum et al., were the first to report on the use of hyperopic-ReLEx and found the procedure to be feasible and effective but lacked stability and predictability [19].

Even with new technology and highly precise treatment nomograms, discrepancies continue to occur between the expected and achieved refractive outcomes [19–21]. A possible explanation for this involves the model of photokeratectomy, which forms the basis of LASIK and photorefractive keratectomy (PRK) ablation profiles [22]. This assumes a biologically and biomechanically inert cornea [23] and does not take into consideration possible alterations due to corneal thickness and biomechanical influences. The lack of studies investigating this relationship and it’s potential impact on corneal biomechanical changes following LASIK for hyperopia, led us to analyse this.

New technology for correcting hyperopia is not available throughout the world, and little is known on the additional complexities this technology presents. Therefore, this study investigated the effects of the amount of treatment, corneal volume, peripheral corneal thickness, residual bed thickness in the periphery, proportion of corneal volume removed and percentage of tissue removed from the corneal periphery on the refractive outcome, three months following LASIK for Hyperopia and Hyperopic/astigmatism.

## Materials and methods

This was a prospective, non-randomised investigation of refractive and corneal topographic outcomes following H-LASIK. Surgery was conducted using a mechanical microkeratome (Hansatome Microkeratome, Bausch & Lomb Surgical, Claremont, CA, USA) and the Technolas 217 C spot scanning Excimer Laser (Bausch & Lomb Surgical, Claremont, CA, USA). Written consent was obtained following explanation of the study procedure. Approval for the studies was obtained from UNSW (the University of New South Wales) Australia Human Ethics Panel (HREA approval number 054005). This research followed the tenets of the Declaration of Helsinki 1983).

This research project was a collaborative initiative between the School of Optometry and Vision Science at UNSW (the University of New South Wales) Australia and the Vision Eye Institute.

Data from 18 eyes of 18 subjects who had undergone H-LASIK were included in this study. Demographic data are shown in Table 1. One eye was selected randomly from each subject. All subjects had a flap thickness of 160 μm and optic zone of 6 mm. Inclusion criteria required a 12-month stable pre-operative refraction. Patients with hard contact lenses discontinued wear for 5 weeks and soft contact lenses for 4 weeks prior surgery to allow the cornea to stabilise. Exclusion criteria included anterior and posterior segment diseases such as keratoconus, dry eye syndrome, corneal infections, systemic immune and collagen deficiencies and rheumatic diseases. Subjects with greater than +5.00 D of hyperopia and/or -5.00 D refractive astigmatism, and insufficient corneal thickness for the procedure (resulting in residual bed thickness of less than 250 μm) were also excluded.

**Table 1:** Subject demographics and outcomes. Spherical equivalent (SE). Dioptres (D). Dioptre sphere (DSph). Location 3mm from the corneal centre along the horizontal axis temporally (3T).

Number of Eyes	18
Males:Females	9:9
Age (Yrs)	50 Range (33 to 62)
Expected Refractive Outcome (D)	Range (0 to -1.75)
Refractive Error (SE D)	+2.00 (range +1.25 to +3.88)
Treatment (SE D)	+2.00 (range 0 to +4.00)
Pulses	1777 (range 576 to 2999)
Total Corneal Volume (mm^3^)	54 (range 41 to 61)
Peripheral corneal Thickness at 3T (μm)	609 (range 537 to 677)
Proportion of Tissue Removed at 3T	8% (range 2 to 17%)
Residual Bed Thickness at 3T (μm)	399 (range 288 to 452)
Proportion of tissue removed (total pulses/ total corneal volume)	56.48 (range 15 to 109)

Pre-operative evaluations included subject history, uncorrected vision, manifest refraction and visual acuity using a Snellen chart at 6m with cycloplegia, slit-lamp biomicroscopy and fundus examination. The Orbscan II Corneal Topography System (Orbscan, Inc., Salt Lake City, UT, USA) was used to obtain three corneal topography maps to assess pachymetry changes.

### Surgical procedure

A standard LASIK technique was used, flap thickness 160μm and optic zone 6mm. Subjects undergoing bilateral LASIK were operated on both eyes at the same visit. Prior to surgery, subjects were given one drop of oxybuprocaine hydrochloride (Minims, 0.4%, Bausch &Lomb, Madison, NJ, USA). Subjects’ eyelashes were draped with an Op-Site drape (Nephew Medical Ltd., Hull, UK) and a wire lid speculum inserted.

The surface of the cornea was irrigated with balanced saline solution (BSS) and a 9.5 mm suction ring placed on the cornea. The corneal flap was cut using the Hansatome Microkeratome (Bausch & Lomb Surgical Claremont, CA, USA). After placing the suction ring on the eye, a tonometer was used to verify that the intraocular pressure was greater than 65 mmHg. The microkeratome head was placed on the suction ring, which advanced and retracted to create the superiorly hinged corneal flap. Suction was released and the instrument removed from the eye. After the flap was lifted the Technolas 217 C Excimer laser (Bausch & Lomb Surgical Claremont, CA, USA) was used to perform the stromal ablation. The laser nomogram was used to determine the depth of tissue ablated and the total number of pulses. Following ablation the flap was repositioned and irrigated with BSS. A protective shield was positioned over the eye until the day-1 aftercare visit. All subjects who had simultaneous LASIK had the procedure performed on the right eye initially followed by the left eye using the same blade.

### Postoperative care

Postoperative medication included antibiotics: Ciloxan (0.3%, Alcon Laboratories (Australia), Frenchs Forest, NSW, Australia and Chlorsig, (0.5%, Sigma Pharmaceuticals (Pty Ltd (Australia), Clayton, Victoria, Australia) qid for 5 days, topical anti-inflammatory: Maxidex (0.1%, Alcon Laboratories (Australia), Frenchs Forest, NSW, Australia) qid for 10 days. Topical lubricants: Refresh Plus lubricant eye drops (0.5% lubricant eye drops Allergan (Australia) Pty Ltd, Gordon, NSW, Australia)) were applied as required.

Post-operative review was performed at 3 months, and included uncorrected visual acuity and best corrected visual acuity (BCVA) following manifest refraction without cycloplegia. Corneal topography was obtained using the Orbscan II Corneal Topography System (Orbscan, Inc., Salt Lake City, UT, USA) and one measurement was taken for each eye. External eye structures were examined by slit-lamp microscopy. To minimise fluctuations in corneal thickness due to diurnal variation, aftercare visits were held later in the day when overnight oedema had subsided and at approximately the same time for each subject. Operative complications including flap complications were noted. Postoperative complications such as inflammation and epithelial ingrowth were also noted.

### Data analyses

**Fig 1:**
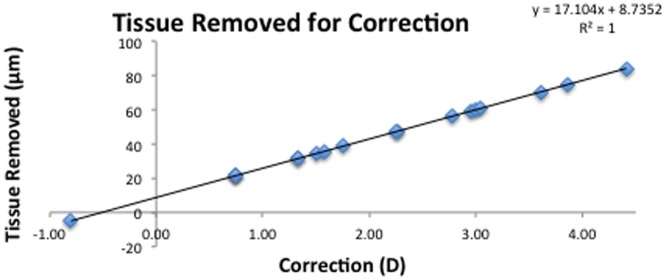
Nomogram for the amount of tissue required to be removed 3mm from the corneal centre to achieve a particular refractive correction.

The Orbscan obtained the following measurements. Corneal volume μm^3^ was calculated using the chord diameter and appropriate volumetric calculation from the Orbscan. Peripheral pachymetry ((3T) (μm), describes corneal thickness at a point 3mm temporal and horizontal to the corneal centre. This location was selected, as it is the point where the maximum ablation depth occurs with a 6 mm optic zone. A graph was produced to determine the maximum depth of ablation for a given correction (Fig 1). Vector analysis was used to determine the correction at 3T and from this information the maximum ablation depth at 3T was obtained. Proportion of tissue removed at 3T was determined as the maximum depth of tissue removed as a percentage of the pre-operative corneal thickness. Proportionate corneal volume removed is the total number of pulses divided by the total corneal volume.

Repeatability of the Orbscan has been well documented in longitudinal and multiple session measurements of corneal thickness [24] and these values were used to determine significant corneal thickness changes.

As subjects undergoing same-session bilateral LASIK demonstrate a strong association in refractive and visual outcomes between eyes [25] we chose one eye per subject randomly for this study. Only subjects who completed all visits were recruited into the study.

The following variables were included in the analyses: age (years) at date of procedure, correction, total number of pulses (obtained from the laser nomogram), corneal volume (determined from the Orbscan), proportion of tissue removed (number of pulses/corneal volume), peripheral corneal thickness at 3T, percentage of tissue removed at 3T, residual bed thickness at 3T. The difference to the expected refraction at three months was the difference between the expected refraction (surgeon’s target refraction) and the achieved refraction.

Associations between the difference to the expected Rx and proportion of tissue ablated at 3T, number of pulses, proportionate corneal volume removed, correction, residual bed thickness at 3T and age were examined using bivariate correlation tests (Pearson’s for normal distribution and Spearman’s for non-normal distribution). Normality was determined by skewness and kurtosis tests. Missing data were excluded pair wise and differences were considered significant at the < 0.05 level. Regression analysis was used to determine the linear relationship between the dependent variable of difference to the expected Rx at 3 months, and the significant independent variables of proportionate tissue ablated at 3T, number of pulses, proportionate corneal volume removed and correction. Linear regression analysis used the stepwise method and only significant independent variables were entered into the analysis.

A sample size of 16 was calculated using a power of 0.80 at the 5% level of statistical significance, and an effect size (f^2^) of 0.587

## Results

### Complications and observations

Epithelial ingrowth, keratectomy and flap complications were not observed and no other vision threatening complications were reported. One subject required an intraocular lens following the procedure due to the presence of a cataract.

### Refractive outcomes at three months

At three months post-surgery the mean spherical equivalent refractive outcome deviation from the expected was -0.20 (range -2.00 - +1.00) DSph. Half of the subjects were overcorrected (9/18).

### Factors associated with the refractive outcome

At three months post-surgery factors significantly associated with the difference to the expected refractive outcome are shown in Table 2 and include the proportion of tissue removed from 3T, the number of pulses required for the refractive treatment, the proportionate corneal volume removed and the total spherical equivalent correction.

**Table 2:** Factors significantly associated with the difference to the expected refractive outcome 3 months post-surgery. The deepest ablation occurs circumferentially 3mm from the corneal centre. 3T refers to a point 3mm from the corneal centre in a horizontal direction temporally. Proportionate tissue ablated at 3T is depth of tissue ablated as a fraction of total corneal thickness at 3T. Proportionate corneal volume removed is the total number of pulses divided by the total corneal volume. Correction (Sph) correction spherical equivalent. Residual bed at 3T is residual bed thickness at 3T.

Factor	n	p-Value	Pearson Correlation
Proportionate tissue ablated at 3T	18	.008	-.605
Number of Pulses	.013	.013	-.574
Proportionate corneal volume removed	13	.003	-.747
Correction (Sph)	18	.016	-.558
Residual bed at 3T	18	.189	.325
Age (yrs)	18	.536	.156

### Difference between the expected and achieved treatment

**Fig 2:**
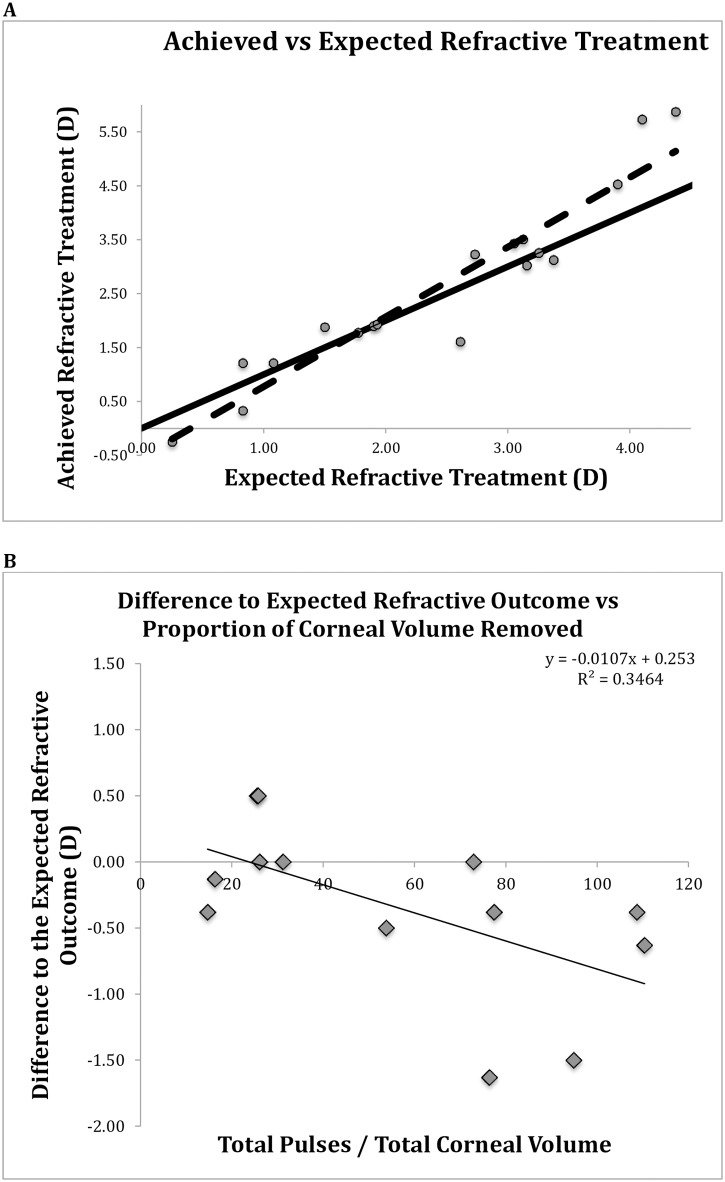
**A**: Three-months post-surgery the mean difference to the expected spherical equivalent refractive outcome was -0.20±0.64 D (range -2.00D to +1.00D). Ideal correction designated with unbroken line. Achieved correction designated with broken line. B: Difference to expected refractive outcome vs proportion of corneal volume removed at 3 months post-LASIK. Dioptres(D). Greater proportion of tissue removed results in overcorrections.

At three months post-surgery patients generally presented with under-corrections when the treatment was less than +2.00 DSph or over-corrections when the treatment was greater than +2.00 DSph (Fig 2A).

### Difference to expected refractive outcome vs proportion of corneal volume removed

At 3 months, patients who had a greater proportion of tissue removed presented with greater overcorrections (Fig 2B)

### Proportion of tissue removed at 3T vs proportion of total tissue removed

**Fig 3:**
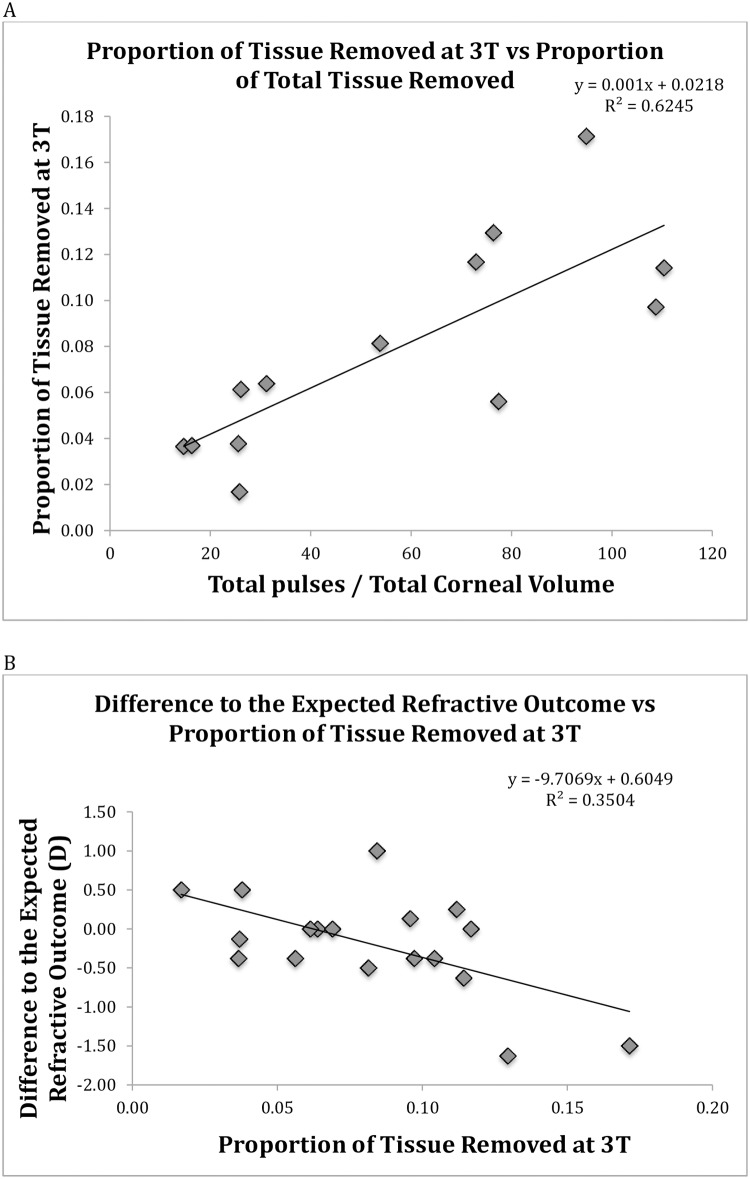
A: Proportion of tissue removed at 3T vs proportion of total tissue removed (total number of pulses/total corneal volume). 3T was determined as the region 3mm from the corneal centre, in a horizontal direction temporally. Proportion of tissue removed at 3T highly correlates with the proportion of total tissue removed from the cornea. **B**: Difference to the expected refractive outcome as a function of proportion of tissue removed at 3T (3mm from the corneal centre temporally along the horizontal axis). Dioptres (D).

Proportion of tissue removed at 3T highly correlates with the proportion of total tissue removed from the cornea (Fig 3A) as with a hyperopic ablation the majority of tissue removed is from 3T.

### Difference to the expected refractive outcome vs proportion of tissue removed at 3T

Patients with greater proportion of tissue removed at 3T presented with higher over-corrections at 3 months (Fig 3B)

### Regression analyses

On linear regression analysis, the percentage of tissue removed at 3T was the single independent significant determinant of the difference to the expected refractive outcome at 3 months post-surgery. Subjects with greater proportion of tissue removed from 3T resulted in greater overcorrections. This variable accounted for 37% (R^2^) of the difference between the achieved and expected refractive outcome at 3 months. The equation predicting the refractive outcome at 3 months is:

Difference to the expected refractive outcome at 3m = 0.65 − (10.21x%3T)

$${\text{R}}^{2}=0.37,\text{ P}=\;.001$$

## Discussion

In this study, at three months, half of the subjects presented with overcorrections up to and including -2.00DSph. This was related to the proportionate volume of tissue ablated, with higher proportions causing greater overcorrections. Although this association is significant, there were 13/18 subjects who had Orbscan results providing total corneal volume. To substantiate this finding we showed a direct correlation between proportionate volume ablated and the proportion of tissue removed at 3T. For this study we used proportion of tissue removed at 3T as a marker of proportionate volume of tissue removed, as there were 18 subjects with this data. We demonstrated that the proportion of tissue removed in the periphery at 3T was strongly correlated with the refractive outcome at three months with greater proportions removed resulting in higher overcorrections. This suggests that hyperopic LASIK potentially causes biomechanical changes, effectively steepening the cornea.

Over or under-corrections following LASIK for hyperopia have previously been reported with 64% of eyes treated being within ±1.0 D, of the intended correction, and 38% of eyes within ±0.50 D, by nine months [19]. Authors concluded that there is an increased tendency toward overcorrections with progressively larger optical zone diameters [20]. Davidorf et al. investigated 3 to 6 month refractive outcomes of subjects undergoing hyperopic LASIK with various optical zone diameters and reported an achieved 112% of the programmed correction in the 6 mm optic zone group. This finding is consistent with the results of the present study, where subjects treated with a 6mm optic zone ended up with a similar range of overcorrections [20].

Refractive outcomes of hyperopic LASIK have been shown to be dependent on the degree and type of hyperopia corrected. Arbelaez et al., reported spherical hyperopia more accurate than toric and low more accurate than high. Subjects with greater corrections showed higher variations from the expected correction [26]. This agrees with our findings, where higher corrections were found to be less precise.

Overcorrections are also observed in hyperopic refractive surgery utilising more recent technology including IntraLase Femtosecond Laser [19, 21, 27, 28]. Interestingly, Gil-Cazorla et al., show better overall refractive results 3 months following surgery when LASIK is performed with the IntraLase femtosecond laser when compared to LASIK utilising a M2 microkeratome [21]. However their results show more eyes (15.28%) overcorrected in the IntraLase group than (4.17%) in the microkeratome group, suggesting the new technology is significantly inferior in minimising overcorrections.

This study was the first to investigate the proportion of tissue removed overall and from the periphery at 3T, as a predictor of the refractive outcomes following hyperopic LASIK. The results confirm that the proportion of tissue removed from 3T significantly predicts the refractive outcome with greater proportions resulting in overcorrections. As this study did not measure corneal biomechanical changes, potentially through hysteresis, we are unable to provide evidence supporting this, however we would like to hypothesize that this refractive outcome may be due to biomechanical changes occurring post-operatively.

Several models have been proposed which describe biomechanical changes occurring to the cornea following refractive surgery interventions [23, 29]. Roberts et al., proposed a biomechanical model that predicts additional flattening over and above whatever ablation profile is programmed, due to peripheral corneal thickening, whether myopic with intent to flatten, hyperopic with an attempt to steepen, or non-refractive phototherapeutic keratectomy [23, 30]. This thickening is believed to be a direct consequence of severed corneal lamellae.

The pre-operative cornea may be conceived as a series of stacked rubber bands (lamellae) with sponges between each layer (inter-lamellar spaces filled with extracellular matrix). The rubber bands are in tension since there is a force pushing on them from underneath intraocular pressure (IOP), and the ends are held tightly by the limbus. After myopic laser refractive surgery, a series of lamellae are severed and obliterated centrally. The remaining peripheral segments relax, just like taut rubber bands would relax once cut. With the reduction in tension in the peripheral lamellae, the squeezing force on the matrix is reduced and the distance between lamellae expands, analogous to the sponges taking up water if the rubber bands are cut. This allows the mid-periphery of the cornea to thicken. Due to the crosslinking between lamellae layers, the expansion force pulls on the underlying intact lamellae. An outward force in the periphery pulls laterally on the centre and flattens it. Thus the cornea will flatten centrally with any procedure that circumferentially severs lamellae. This includes myopic profiles, hyperopic profiles, constant depth PTK profiles, as well as the simple cutting of a LASIK Flap [23].

Roberts et al., proposed that even the process of cutting a flap for LASIK would be enough to cause biomechanical changes to the cornea [23]. However, investigations have found that the initial effect of the keratome cut alone on the human cornea has limited biomechanical effects. Initially, the cut causes microfolds in the stromal flap and highly reflective debris particles to form on the interface. These effects remain unchanged 1 month after the cut, which indicates that the keratome cut alone causes minimal changes [31].

Further studies however have shown that biomechanical alterations do occur in response to cutting a flap. Knox Cartwright et al., investigated the effects of varying flap depth and side cut angulations in LASIK and demonstrated weakening of corneal strength that was related to the depth of the cut, with deeper flaps and vertical side cuts contributing to loss of corneal structural integrity [32]. Marshall et al. also support this, by showing that the strongest part of the cornea is located in the midperiphery, the interior part of the cornea is progressively weaker than the surface due to the anterior lamellae contributing to the majority of corneal strength and 75% of weakened corneal biomechanics is attributed to the side cut and not from lamellar bed cuts [33].

Clinical evidence for this phenomenon has been reported in the literature, where primary hyperopic procedures require treatments of up to 35% greater than secondary hyperopic procedures (hyperopia resulting from an overcorrected myopic refractive procedure) [34]. This suggests the mid-periphery of a primary hyperopic procedure thickens following surgery possibly due to biomechanical changes, compared to secondary hyperopia, where the integrity of the cornea, already compromised by the previous surgery, does not. This would theoretically make hyperopia a more difficult procedure since the biomechanical flattening would be in opposition to the ablation profile, producing under-corrections and thus requiring a deeper ablation to offset the biomechanical response. This model would substantiate data in this study associated with lower proportion of tissue removed at 3T (corrections less than +2.00DSph), as these subjects were under-corrected suggesting a slight reversal of the refractive procedure with thickening in the mid-periphery and flattening in the centre. Data from this study is also supported by Davidorf et al., who found subjects undergoing LASIK for hyperopia were generally under-corrected when treatments were less than +3.00 DSph and 5mm optic zones [20].

In a hyperopic ablation, minimal tissue is removed from the centre and progressively more tissue is removed towards the mid-periphery with the greatest amount of tissue removed 3mm from the corneal centre for a 6mm optic zone. In this study, we investigated the impact that the proportion of tissue removed 3mm peripherally along the horizontal axis temporally (3T) has on the refractive outcome and we found that when this was high, treatments exceeding +2.00 DSph correction, it resulted in overcorrections. This study demonstrates that subjects with thicker corneas will have better refractive predictability, particularly in greater corrections. Although not measured in this study, one possible explanation for this effect, that will need investigation in future studies may be due to biomechanical changes causing central corneal steepening over time or a reversal of initial biomechanical changes induced by the procedure.

**Fig 4:**
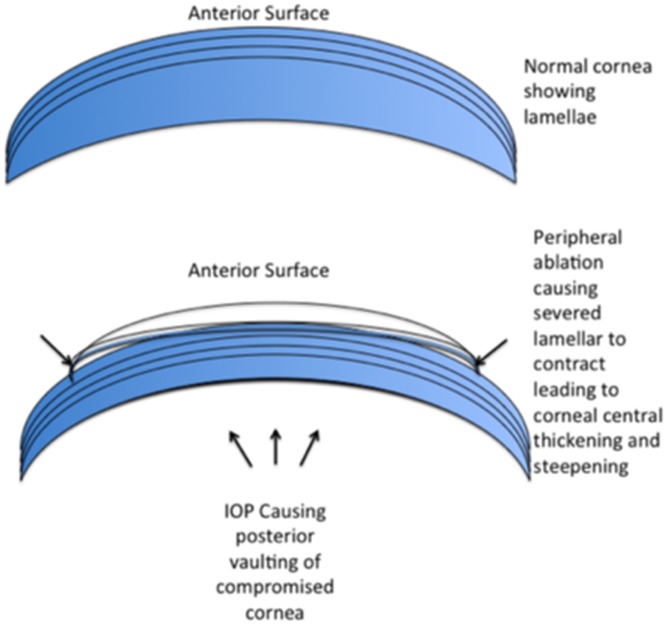
Schematic representation of potential biomechanical changes to the cornea in response to hyperopic laser in situ keratomileusis. The lamellae severed in the periphery, are no longer under tension from their connection to the limbus and therefore contract increasing the corneal central thickness and steepening the cornea. The posterior corneal surface of the compromised cornea vaults anteriorly from the intra ocular pressure altering the corneal power.

The cornea is composed of lamella containing highly oriented arrays of collagen fibrils. The lamellar size varies considerably as a function of depth in the stroma with the smaller anterior lamellae measuring 0.5–30 μm wide and 0.2–1.2 μm thick and the larger posterior ones measuring 100–200 μm wide and 1.0–2.5μm thick [35]. It would therefore be possible for ablations removing greater proportions of tissue to sever deeper larger lamellae. With no peripheral anchoring these lamellae are free to contract centrally causing thickening of the corneal centre resulting in overcorrections (Fig 4).

Our hypothesis is potentially supported by Liu et al., who used transmission electron microscopy to evaluate ultrastructural changes in the corneal centre following myopic and hyperopic SMILE procedures in an animal model [36]. They showed a less distinct border between the laser-disrupted tissue and surrounding collagen fibres and the collagen fibres appeared more deranged in the hyperopic-SMILE group when compared to the myopic-SMILE group. These results suggest the anterior lamellae of the hyperopic ablation, with no peripheral anchoring contract and therefore become more deranged than those observed in the myopic ablation where the central fibres are ablated and the peripheral remain anchored.

These findings are also supported by Davidorf et al., who found larger optic zones, with more peripheral ablations result in greater overcorrections throughout the entire range of treatments suggesting longer lamellae contracting centrally produce more central corneal thickness and subsequent overcorrections [20]. Furthermore, Chen et al., provide evidence that overcorrections are caused by corneal biomechanical changes, steepening the cornea more than intended [37]. The authors showed overcorrections (-0.37± 0.50) following hyperopic LASIK were associated with an increase in central corneal curvature (preoperative 43.12±1.13 –postoperative 44.79±1.20).

Another explanation for the overcorrections seen in this study could be due to weakening of the cornea causing central corneal ectasia. Randleman et al., showed that corneal ectasia occurs following hyperopic LASIK in patients with both normal and abnormal preoperative topography [38]. In normal subjects there is a significant but weak correlation between central corneal thickness and intraocular pressure (IOP). Greater corneal thickness is associated with higher IOP [39]. Any changes therefore to corneal thickness would influence IOP. This is critical in subjects undergoing refractive surgery, as the measured IOP may not be indicative of the actual IOP. In hyperopic patients, IOP appears lower after LASIK, the applanation tonometer underestimating the true IOP [40]. This reduction is not correlated to the amount of tissue removed or to flattening or steepening of the central cornea [41]. This apparent lower IOP suggests the cornea becomes more pliable following LASIK. A more pliable cornea, with the same IOP, could result in corneal steepening through a type of iatrogenic ectasia. Although not measured in this study, we hypothesize that this corneal steepening could therefore be responsible for the overcorrections found in this study at three months and may be attributed to instantaneous biomechanical changes in which IOP pushes against the back surface of a surgically compromised cornea [29]. Refer to Fig 4 for a schematic representation.

A new procedure combining LASIK and corneal cross-linking (Xtra) has been investigated by Rajpal et al [42]. They showed LASIK Xtra to be superior in refractive stability when compared to the standard LASIK procedure for both myopic and hyperopic patients and in particular the higher corrections that are more at risk of refractive drift from the intended correction. This suggests corneal instability might partially be responsible for the refractive variations observed post-LASIK.

Corones et al., noted in their study ablation algorithms incorporated overcorrections to overcome expected regression due to wound healing [43]. Intended overcorrection could in part explain the results observed in this study, however subjects were not intentionally overcorrected and the range of refractive outcomes observed was too big to be explained simply by an overcorrection. If subjects in this study were intentionally overcorrected, then all subjects would have shown overcorrection, which was not the case.

Although the post-LASIK process of wound healing is complex, the cornea has the ability to recover quite effectively and rapidly from LASIK. Many processes responsible for corneal clarity including the epithelium and endothelium and a healthy interaction between stromal structural components such as collagen and proteoglycans are compromised following LASIK leading to transient corneal oedema. Corneal clarity is maintained through the endothelium pump [44]. To maintain this clarity the endothelium needs to be both healthy and sustain minimum numbers. Acute corneal endothelial changes observed within 15 minutes of LASIK surgery have been shown to resolve as early as the next day [45]. Corneal endothelial cell count continued to remain consistent up to 12 months following the procedure [46], demonstrating endothelial safety following LASIK.

Liu et al., comprehensively discussed the healing profiles following hyperopic refractive surgery in an animal model [36]. They compared the wound healing process between hyperopic-small incision lenticule extraction (SMILE) and LASIK. In vivo confocal microscopy was used to evaluate the corneal stromal reaction and keratocyte activation. Stromal reflectivity observed 4 days post-operatively at the cap or flap interface subsided in all eyes by week 4. The expression of CD11b (inflammatory marker) was not significant in all eyes at 1 or 4 weeks post-operatively. Fibronectin produced by activated stromal fibroblasts and shown to play a significant role in the corneal wound healing process, was less distinct at 4 weeks, suggesting the healing process nearing completion. Heat shock protein 47 (HSP47) is a protein that responds to stress and has a pro-fibrogenic role in the wound healing process as well as an inducer of collagen production in keratocytes [47]. This was found to be upregulated in the LASIK eyes showing activation of this process. Although HSP47 was detected in keratocytes up to day three it was not observed after that in PRK. Taken together these finding suggest that the cornea would have mostly healed by three months when data for this study was collected.

The nomograms used for LASIK are calculated in terms of empirical population data on anterior corneal surface changes and do not take into consideration individual biomechanical changes to the posterior corneal surfaces. Early studies of posterior corneal surface topographic changes after myopic LASIK have shown an increase in the posterior corneal elevation that correlates significantly with the attempted correction, post-operative pachymetry, pre-operative central pachymetry, residual bed thickness and amount of ablation [48–51]. Similar elevation may be partly responsible for the overcorrections found in this study. As investigations of posterior corneal elevation following LASIK are still in the experimental stages, and the accuracy and repeatability of Orbscan difference maps used for analyses not yet known, we did not record this data.

Finally, evidence supports the understanding that central corneal thickness has a strong genetic component [52]. Twin studies have shown central corneal thickness is one of the most highly heritable human traits, whilst other studies have shown clear ethnic-related differences in corneal thickness. The genetic components responsible for this remain to be elucidated, however a better understanding of this might benefit the field of refractive surgery, similarly to the understanding realized in glaucoma [53]. Taken together, a better understanding of the genetics responsible for corneal thickness and the proportion of peripheral tissue removed could potentially be used in the future to promote a more accurate outcome with less regression.

As all studies, this one has limitations and flaws. Firstly, although our statistical analysis suggested we required a minimum sample size of 16, the 18 subjects used were still a small number providing a limitation to this study.

Corneal thickness can be measured using a variety of instruments. Ultrasound pachymetry (US pachymetry) utilises a contact method and is perhaps the most commonly used. However non-contact methods such as the scanning slit topography, Orbscan II (Bausch & Lomb, Rochester, NY, USA) is also used. Recently, Khaja et al., compared and correlated central corneal thickness (CCT) measurements determined by US pachymetry with Orbscan and reported that in subjects with healthy corneas, there was a significant linear correlation (r2 = 0.96, P<0.0001) between the instruments [54].

US pachymetry is considered the current gold standard for measuring CCT as previous studies have reported the Orbscan consistently measuring 30–50 μm thicker [55]. Most recent studies however have shown CCT measures only 3μm thicker in Orbscan than US pachymetry, demonstrating a more accurate result than previously thought [54].

The Orbscan II (Bausch & Lomb, Rochester, NY, USA) was chosen to measure corneal thickness *in vivo* for these studies as it can simultaneously measure corneal topography and corneal thickness and allows accurate location of corneal positions. Although US pachymetry might provide more accurate results in some cases, it would not have allowed for the pinpoint accuracy required in this study to measure corneal thickness 3mm from the centre repeatedly, thus resulting in less repeatable measurements. In this study, we were interested in thickness changes using the Orbscan II, not accuracy. Therefore taking repeated measurements at the same locations, with the same instrument and using the difference between these measurements we were able to determine the change in corneal thickness between pre-operative and post-operative time points.

An important finding of this study is the potential biomechanical changes induced to the cornea by H-LASIK. A limitation of this study is that we did not measure any biomechanical changes to the cornea. We can only hypothesize that this occurs and we are unable to provide evidence.

### Conclusion

Refractive ablation nomograms for hyperopia have not taken into account the proportion of corneal volume removed or the proportion of tissue removed in the mid-periphery (3T). They have used empirical population data and modified Munnerlyn formulae in an attempt to predict the refractive outcome. This study shows that the proportion of tissue removed from the mid-periphery has a strong association with the refractive outcome and future refractive nomograms should take this into consideration when determining treatments, particularly for higher attempted corrections. The findings of our study are relevant even in case of newer technologies used for similar purposes. Future researchers are encouraged to use similar investigations as presented in the current Study, utilising the new technology available in an effort to improve those outcomes.
